# Sex-dependent effects of multiple acute concurrent stresses on memory: a role for hippocampal estrogens

**DOI:** 10.3389/fnbeh.2022.984494

**Published:** 2022-09-08

**Authors:** Rachael E. Hokenson, Yasmine H. Alam, Annabel K. Short, Sunhee Jung, Cholsoon Jang, Tallie Z. Baram

**Affiliations:** ^1^Department of Anatomy/Neurobiology, University of California, Irvine, Irvine, CA, United States; ^2^Department of Biological Chemistry, University of California, Irvine, Irvine, CA, United States; ^3^Department of Pediatrics, University of California, Irvine, Irvine, CA =, United States; ^4^Department of Neurology, University of California, Irvine, Irvine, CA, United States

**Keywords:** stress, memory, estrogen, aromatase, hippocampus, PTSD, ELISA, mass spectrometry

## Abstract

Memory disruption commonly follows chronic stress, whereas acute stressors are generally benign. However, acute traumas such as mass shootings or natural disasters—lasting minutes to hours and consisting of simultaneous physical, social, and emotional stresses—are increasingly recognized as significant risk factors for memory problems and PTSD. Our prior work has revealed that these complex stresses (concurrent multiple acute stresses: MAS) disrupt hippocampus-dependent memory in male rodents. In females, the impacts of MAS are estrous cycle-dependent: MAS impairs memory during early proestrus (high estrogens phase), whereas the memory of female mice stressed during estrus (low estrogens phase) is protected. Female memory impairments limited to high estrogens phases suggest that higher levels of estrogens are necessary for MAS to disrupt memory, supported by evidence that males have higher hippocampal estradiol than estrous females. To test the role of estrogens in stress-induced memory deficits, we blocked estrogen production using aromatase inhibitors. A week of blockade protected male and female mice from MAS-induced memory disturbances, suggesting that high levels of estrogens are required for stress-provoked memory impairments in both males and females. To directly quantify 17β-estradiol in murine hippocampus we employed both ELISA and mass spectrometry and identified significant confounders in both procedures. Taken together, the cross-cycle and aromatase studies in males and females support the role for high hippocampal estrogens in mediating the effect of complex acute stress on memory. Future studies focus on the receptors involved, the longevity of these effects, and their relation to PTSD-like behaviors in experimental models.

## Introduction

Studies on the effects of stress on memory have largely centered on chronic stress, which is well established to disrupt hippocampal memory (Kleen et al., 2006; Peay et al., 2020). Whereas acute stress generally enhances memory (Uysal et al., 2012; Brivio et al., 2020), acute traumatic events composed of simultaneous physical, emotional, and social stresses are increasingly recognized to provoke stress-related mental illnesses, including PTSD, and associated spatial memory impairments (Lawyer et al., 2006; Cherry et al., 2010, 2011; Millan et al., 2012; Tempesta et al., 2012; Lowe and Galea, 2017; Lowe et al., 2017; Musazzi et al., 2017; Novotney, 2018; Bell et al., 2019; Harrison, 2019). Additionally, sex differences in stress-related disorders are pronounced, with women generally having higher rates or more severe symptoms (Christiansen and Hansen, 2015; Olff, 2017).

We have previously discovered that exposure to simultaneous short stresses [multiple concurrent acute stresses (MAS)], impairs spatial memory and disrupts thin dendritic spines in hippocampal CA1 in male rodents (Chen et al., 2008, 2010, Chen et al., 2016; Maras et al., 2014). Interestingly, in female mice, the impacts of MAS are estrous cycle dependent. Surprisingly, MAS impair memory when mice are stressed during early proestrus, when levels of estrogens are high. Notably, spatial memory was protected from MAS during estrus, characterized by lower estrogens (Hokenson et al., 2021).

Proestrus-selective stress-induced memory disruptions were unexpected. While estrogens can modulate stress hormone release (Viau and Meaney, 1991; Lund et al., 2006; Heck and Handa, 2019), estrogens are neuroprotective from stress (Wei et al., 2014; Luine, 2016; Azcoitia et al., 2019). Others reported that higher estrogen levels associate with deleterious effects of stress (Shors et al., 1998; Gupta et al., 2001; Rubinow et al., 2004; Shansky et al., 2004, 2009). These disparate findings suggest a nuanced role of estrogens, where their interactions with stress and memory are likely influenced by dose, origin, interaction with other hormones, stressor type, and the brain regions and networks involved (Holmes et al., 2002; McLaughlin et al., 2008; Barha et al., 2010; Babb et al., 2014; Korol and Pisani, 2015; Graham and Daher, 2016; Graham and Scott, 2018; Cohen et al., 2020; Duong et al., 2020).

Given the profound effects of MAS on hippocampus (Chen et al., 2008, 2010, Chen et al., 2013, 2016; Maras et al., 2014; Hokenson et al., 2021), the important role of estradiol in learning and memory for males and females (Frick et al., 2015, 2018; Luine et al., 2018; Chen et al., 2021) and because hippocampal estradiol levels are reported to be higher in male and proestrus female compared to estrous female rats (Hojo et al., 2004; Kato et al., 2013; Hojo and Kawato, 2018), we hypothesized that high levels of hippocampal estradiol are required for, and perhaps mediate, MAS-provoked memory impairments in both male and female mice. Here we tested this notion by blocking aromatase, an enzyme required for the production of estrogens, for 1 week leading up to MAS. This blockade prevented MAS-induced spatial memory deficits in male and female mice, supporting a deleterious role of estrogens in MAS-induced memory impairments.

We sought to quantify circulating and hippocampal estradiol levels to confirm reported levels by sex and cycle (Hojo et al., 2009; Kato et al., 2013; Hojo and Kawato, 2018) and to assess the efficacy of aromatase inhibition. We identified apparent reductions in the serum and hippocampal estradiol-immunoreactivity in mice treated with aromatase inhibitors, but analyses by mass spectrometry indicate that the compound measured using ELISA might not be estradiol.

## Materials and Methods

### Animals

C57BL/6J 2–5-month-old virgin male and female mice were purchased from Jackson Laboratories or bred in-house. Two to five same-sex mice were group housed in individually ventilated cages (Envigo 7092-7097 Teklad corncob bedding, iso-BLOX^TM^ nesting) and had *ad libitum* access to water and food (Envigo Teklad 2020x global soy protein-free extruded). The vivarium was maintained between 22 and 24°C on a 12-h light/dark cycle (lights on 6:30 a.m.). Stress and behavior tests occurred during the light phase.

### Multiple concurrent acute stresses (MAS)

**Figure 1:**
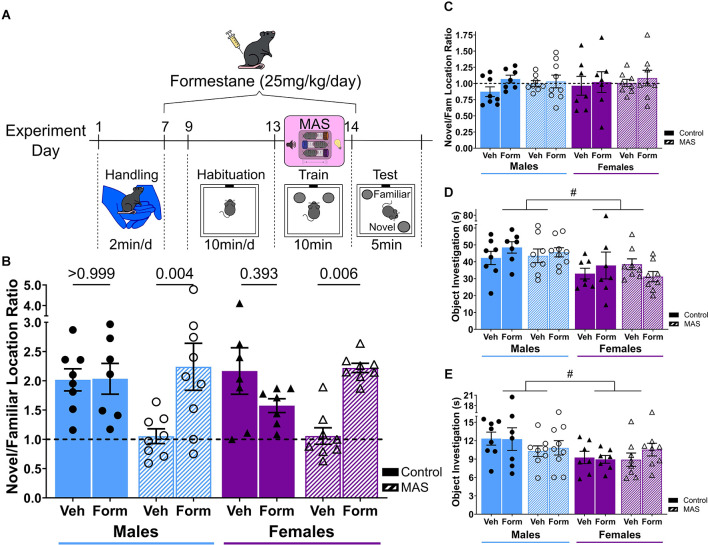
Aromatase inhibition prevents MAS-induced object location memory deficits in male and female mice. **(A)** Mice were handled, then given daily, subcutaneous administrations of the aromatase inhibitor formestane for 1 week leading up to MAS, concurrently with habituation to the object location memory (OLM) apparatus. Two hours after MAS, mice were trained with two identical objects for 10 min then tested 24 h later. **(B)** During the 5 min OLM test, vehicle treated male and proestrus female mice exposed to MAS had poor spatial memory compared to controls, however, male and female mice treated with formestane prior to MAS preferentially explored the moved object (*n* = 7–9/group). **(C)** During OLM training, mice did not display any object bias regardless of group assignment. **(D)** During OLM training, time spent investigating the objects did not vary with drug or stress, but males had longer total investigation compared to females. **(E)** Likewise, during OLM testing, time spent investigating the objects did not vary with drug or stress, but males had longer total investigation compared to females. ^#^Main effect (*p* < 0.05). Post test p-values are provided above the corresponding comparisons. Individual points represent individual mice. Data are presented as mean ± SEM.

Male and female mice were assigned to a home-cage (“unstressed”) control or a multiple concurrent acute stresses (MAS) group (Figure 1A). Two to nine mice were individually restrained in a ventilated 50 ml plastic tube and jostled on a laboratory shaker in a room bathed with loud (90 dB) rap music and bright lights for 2 h (Hokenson et al., 2020). Behavioral tests were conducted 2 h post cessation of MAS, when plasma corticosterone and total object exploration time is equivalent between stressed and control animals (Maras et al., 2014; Chen et al., 2016; Hokenson et al., 2021).

### Estrous cycle monitoring

Estrous cycle phases were monitored daily *via* vaginal cytology. Cells were stained with the Shandon Kwik-Diff Kit (Thermo Fisher 9990700) and cycle phases were classified based on relative cell type composition (Byers et al., 2012; McLean et al., 2012; Hokenson et al., 2021). To account for the potential effects of daily smearing on behavior, male mice were “faux” smeared with a cotton swab (Sava and Markus, 2005). Smears were taken during the morning alongside the administration of formestane/vehicle, 1 h prior to MAS.

### Pharmacology

**Figure 2:**
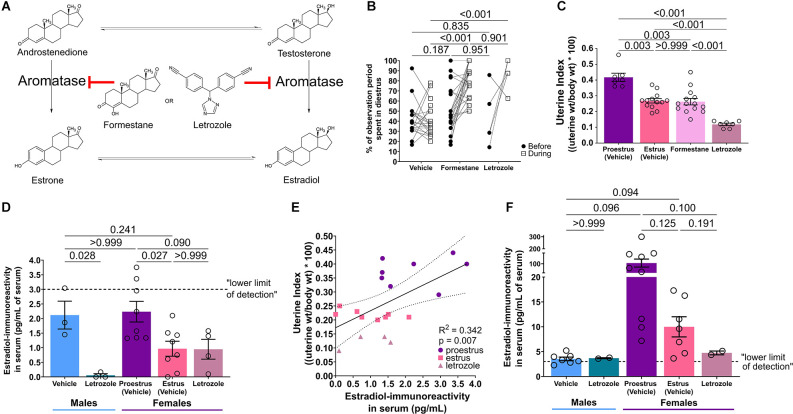
Aromatase inhibition disrupts estrous cycling and decreases systemic estradiol levels. **(A)** Aromatase inhibitors, formestane and letrozole, block the conversion of androgens to estrogens. **(B)** During treatment, aromatase inhibitors increase the percentage of time the female mouse spends in the diestrus phase of the cycle (*n* = 7–24/group). **(C)** Uterine indices are decreased in estrus compared to proestrus mice. Treatment with aromatase inhibitors likewise decreases uterine indices (*n* = 7–14/group). **(D)** Estradiol-immunoreactivity was quantified in serum directly measured by ELISA (unextracted). Letrozole treatment tended to decrease estradiol levels and proestrus levels were elevated compared to estrus. However, male vehicle serum estradiol levels were high, similar to proestrus female levels. Most values are below the lower limit of detection of the assay (*n* = 3–8/group). **(E)** There is a positive correlation between estradiol-immunoreactivity in serum measured by ELISA and uterine index in female mice (*n* = 20). **(F)** Estradiol-immunoreactivity was additionally quantified in serum that was extracted prior to ELISA. Again, letrozole treatment tended to decrease estradiol levels and proestrus levels were elevated, though with even larger variability, compared to estrus. With extraction, male serum estradiol levels were below female (*n* = 2–9/group). Post test p-values are provided above the corresponding comparisons. Individual points represent individual mice and matched points represent a mouse at different time points. Data are presented as mean ± SEM.

The conversion of androgens to estrogens (Figure 2A) was blocked with subcutaneous administration of the steroidal, aromatase inactivator formestane (4-Hydroxyandrost-4-ene-3,17-dione, Sigma-Aldrich F2552; Yue et al., 1995; Nißlein and Freudenstein, 2007) at 25 mg/kg/day (or corn oil vehicle) each morning for 8 days, with the final dose given 1 h before MAS. Treatments were assigned randomly but per cage to avoid cross-contamination. Formestane cross-reacted with our estradiol ELISA (a concentration analogous to max expected levels was interpreted as having >200 pg/ml estradiol), thus for estradiol quantification in serum and hippocampus we employed mice treated intraperitoneally with the non-steroidal inhibitor letrozole [4,4’-(1H-1,2,4-triazol-1-ylmethylene) *bis*-benzonitrile, 2 mg/kg/day (Kafali et al., 2004)] or vehicle (1% DMSO in saline). Only formestane was used for behavioral studies. Corn oil and 1% DMSO in saline vehicle treated mice were pooled for analyses of time in diestrus and uterine index.

### Object location memory (OLM)

For OLM (Vogel-Ciernia and Wood, 2014; Figure 1A), mice (*n* = 7–9/group) were handled (2 min/day, 6 days) then habituated to an empty apparatus (10 min/day for ≥5 days) leading up to MAS. Mice were trained (2 h after MAS) for 10 min with two identical objects. In the 5-min testing session 24 h later, one object was displaced while the other remained in the same location (counter-balanced). Object investigation was scored using BORIS version 7 (Friard and Gamba, 2016) by two independent observers unaware of the experimental conditions and was defined as the mouse’s nose pointed ≤1 cm toward the object. Performance is expressed as the ratio of time spent exploring the object in the novel vs. the familiar location (a ratio of 1.0 indicates no preference). Two mice were excluded for under exploration (<5 s during testing), one mouse was excluded due to object bias during training (a ratio >2.0), one mouse was excluded for incorrect cycle phase, and two mice were excluded for being statistical outliers.

### Tissue collection

#### Uterus dissection

Uteri, whose weights fluctuate with cycle and estrogen manipulation (Yue et al., 1995; Zysow et al., 1997; Zhou et al., 2010; Xiao et al., 2018), were weighed (wet weight) and normalized to body weight by computing a “uterine index” {[uterine weight (g)/body weight (g)] × 100; *n* = 7–14/group; Hokenson et al., 2021}.

#### Serum and fresh-frozen hippocampus

Mice were euthanized by rapid decapitation. Trunk blood was collected (within 1–2 min), clotted for 30 min (RT), centrifuged (1,100 *g*, 15 min), then the clear supernatant (serum) was collected and stored at −20°C. For hippocampi, brains were immediately removed from the skull. Hippocampus was dissected on ice (2 min), flash frozen on dry ice, weighed, then stored at −80°C.

### Tissue extraction

To remove interfering substances and enhance estradiol signal, serum and hippocampi were extracted. 100 μl thawed serum was extracted twice (5:1 ratio diethyl ether: serum). After 30 min, samples were frozen in a methanol/dry ice bath and the organic (unfrozen) phase was transferred to a new glass tube, dried, then stored until analysis (Krentzel et al., 2020; Proaño et al., 2020). Hippocampi (20 mg) were processed using liquid-liquid and solid-phase extraction (Chao et al., 2011; Tuscher et al., 2016). Fresh frozen hippocampus was homogenized in 250 μl ice-cold 0.1 M phosphate buffer (PB) *via* pestle (Zymo H1001). Ether extractions (repeated three times) were performed by adding 375 μl diethyl ether, vortexing (30 s), centrifuging (10,000 *g*, 10 min, 4°C), and incubating in a methanol/dry ice bath. The organic phase (unfrozen) was transferred to a glass culture tube and dried (50°C water bath). 100% methanol: dichloromethane (1:1) was dripped into the tubes and evaporated under an airstream. For solid-phase extractions, solvents were eluted through C18 columns (Empore^TM^ Extraction Cartridge C18-SD 3 ml, Supelco 66872-U) with positive pressure (adapter Supelco 57020-U). Columns were first conditioned [250 μl 100% methanol, then 250 μl double-distilled water (×2)]. Samples were eluted (resuspended in 250 μl of 0.1 M PB), washed (×2 250 μl double-distilled water), and two organic elutions (250 μl 100% methanol) were collected. Organic layers were evaporated under airstream/50°C water bath. Methanol: dichloromethane was again dripped into tubes and evaporated under an airstream. Dried samples were stored at −20°C. An estradiol control was run during each extraction round to calculate recovery (99% ± 14%).

### Estradiol enzyme-linked immunosorbent assay (Estradiol ELISA)

Estradiol-immunoreactivity (estradiol-IR) was quantified by the Calbiotech Mouse/Rat Estradiol ELISA Kit (ES180S-100, Supplementary Table S1). Samples (duplicates) were compared to a standard curve generated by the provided standards. Dried extracted serum (*n* = 2–9/group) or hippocampus (*n* = 3–13/group) were resuspended in 1% BSA in 1× PBS [(Silva et al., 2013) and correspondence with the manufacturer], a buffer found to have little interference with the assay. Some serum (*n* = 3–8/group) was dispensed directly onto the plate without extraction as per the manufacturer’s instructions. Absorbances were read (450 nm) with a microplate reader (BioTek^®^ Synergy HTX). Concentrations are expressed as estradiol-IR (picograms) relative to grams hippocampus wet weight or serum volume after correction for recovery.

### Mass spectrometry

Dried extracted hippocampi (*n* = 2–7/group) were resuspended in isopropanol. Thermo Q Exactive Plus Hybrid Quadrupole-Orbitrap Mass Spectrometer coupled with Vanquish UHPLC system was used. LC-MS system was controlled by Xcalibur software (Thermo). Metabolite separation was conducted by Xbridge BEH amide column (2.1 × 150 mm, 2.5 μm particle size, 130 A° pore size; Waters, Milford, MA). LC gradient was generated using LC solvents (solvent A) 20 mM ammonium acetate, 20 mM ammonium hydroxide in 95:5 acetonitrile: water, pH 9.45; (Solvent B) acetonitrile. The chromatography gradient of solvent A and solvent B was run at a flow rate of 150 μl/min: 0 min, 90% B; 2 min, 90% B; 3 min, 75% B; 4.5 min, 0% B; 6 min, 0% B; 7 min, 90% B; 9 min, 90% B; 10 min, 90% B. Autosampler temperature was set to 4°C and column temperature to 25°C. MS analysis was performed with a full-scan mode for measurement of samples (m/z range 260–280, negative ion mode). Tissue sample extracts were compared to standards: 17β-estradiol (Cayman 10006315), 17α-estradiol (Cayman 20776), and 17β-estradiol-d_2_ (Cayman 9002846) dissolved in isopropanol. To obtain MS/MS spectra for estradiol and hippocampus peak, a Targeted Selected Ion Monitoring (Targeted SIM) mode coupled with a data-dependent MS/MS (dd-MS2) scan was used. SIM scans were acquired based on the inclusion of the parent ion (271.1704 m/z) with a normalized collision energy (NCE) of 80. MS/MS spectra were then collected at a resolution of 70,000 (271.1704 m/z) with an automatic gain control (AGC) target value of 1 × 106 and maximum fill times of 100 ms. Hippocampi were first spiked with 17β-estradiol-d_2_ prior to extraction and raw ions of measured compound were adjusted to correct for recovery (69% ± 4%) and then normalized to hippocampus weight.

### Statistical analyses

Analyses employed GraphPad Prism v9.3.1 (Windows). Behavioral data were analyzed with 3-way ANOVA, with factors of sex, drug, and MAS. Time in diestrus was analyzed with 2-way repeated measures ANOVA with drug and time as factors. Ordinary one-way ANOVA was used to analyze estradiol concentrations. Brown-Forsythe ANOVA tests were used when population standard deviations differed. If an interaction was statistically significant (*α* = 0.05), *post-hoc* tests with Sidak’s multiple comparisons (or Dunnett’s T3 for Brown-Forsythe ANOVA) were performed. For estradiol quantification, planned comparisons to compare across sex and cycle or to examine the effects of the drug within sex were employed. The correlation between estradiol-IR and uterine index was computed with Pearson’s correlation. Outliers were excluded by ROUT when applicable. Data are presented as means ± SEM.

## Results

### Aromatase inhibition with formestane protects spatial memory from MAS in male and female mice

To test the potential role of estrogens in the effects of MAS on object location memory (OLM), aromatase was inhibited in male and female mice for 1 week leading up to stress (Figure 1A). Male mice exposed to MAS or female mice experiencing MAS during early proestrus had poor spatial memory when compared to controls (Figure 1B) as previously described (Chen et al., 2016; Hokenson et al., 2021). Treatment with the aromatase inhibitor formestane protected memory in both sexes (Figure 1B). Three-way ANOVA showed an effect of drug (*F*_(1,54)_ = 6.28, *p* = 0.015) and an interaction of drug × MAS (*F*_(1,54)_ = 17.0, *p* < 0.001). There were no effects of sex (*F*_(1,54)_ = 0.21, *p* = 0.649) or MAS (*F*_(1,54)_ = 2.98, *p* = 0.090), nor interactions between drug × sex (*F*_(1,54)_ = 0.80, *p* = 0.376), sex × MAS (*F*_(1,54)_ = 0.17, *p* = 0.683), or drug × sex × MAS (*F*_(1,54)_ = 0.69, *p* = 0.411). *Post-hoc* testing indicated a difference in performance between vehicle MAS males and formestane MAS males (*t*_(54)_ = 3.51, *p* = 0.004) and a difference between vehicle MAS (proestrus) females and formestane MAS females (*t*_(54)_ = 3.35, *p* = 0.006). There were no differences between vehicle control males and formestane control males (*t*_(54)_ = 0.05, *p* > 0.999) or vehicle control (proestrus) females and formestane control females (*t*_(54)_ = 1.59, *p* = 0.393, Supplementary Table S2).

Notably, differences in OLM were not attributed to differences in object exploration or bias during training. During training, the ratio of time spent exploring the object to be moved over the familiar object did not differ across groups (Figure 1C, Table 1). There was an effect of sex on total object investigation time during both the training and testing sessions (Figures 1D,E, Table 1), however, there were no effects of drug or stress on exploration time (Table 1).

**Table 1:** Three-way ANOVA results of object bias during training and objection investigation times during training and testing.


**Object bias during training session**			

**Factor**	**F**	**DFn, DFd**	***P* value**

Drug	1.59	1,54	0.212
Sex	0.13	1,54	0.719
MAS	0.46	1,54	0.501
Drug × Sex	0.11	1,54	0.745
Drug × MAS	0.27	1,54	0.608
Sex × MAS	0.003	1,54	0.957
Drug × Sex × MAS	0.42	1,54	0.520

**Object investigation time during training session**			

**Factor**	**F**	**DFn, DFd**	***P* value**

Drug	0.25	1,54	0.620
Sex	11.6	1,54	0.001
MAS	0.05	1,54	0.816
Drug × Sex	0.87	1,54	0.355
Drug × MAS	1.97	1,54	0.166
Sex × MAS	0.003	1,54	0.959
Drug × Sex × MAS	0.47	1,54	0.494

**Object investigation time during testing session**			

**Factor**	**F**	**DFn, DFd**	***P* value**

Drug	0.32	1,54	0.577
Sex	6.30	1,54	0.015
MAS	0.49	1,54	0.486
Drug × Sex	0.08	1,54	0.785
Drug × MAS	0.64	1,54	0.426
Sex × MAS	2.16	1,54	0.148
Drug × Sex × MAS	0.17	1,54	0.684

Together these results replicate our prior findings that MAS impair spatial memory in male and early proestrus female mice. Blocking the production of estrogens with an aromatase inhibitor leading up to MAS protects OLM in males and females, suggesting that high levels of estrogens are required for MAS to disrupt spatial memory.

### Aromatase inhibition disrupts cycling and decreases systemic estradiol levels

One week of aromatase inhibition with inhibitors formestane or letrozole disrupted estrous cycling in female mice, pausing cycles in the diestrus phase (low estrogens; McLean et al., 2012). The percent of the observation period the mouse spent in diestrus was increased during aromatase inhibition compared to vehicle (Figure 2B). Two-way repeated measures ANOVA identified an interaction of drug × duration of diestrus (*F*_(2,53)_ = 9.25, *p* < 0.001), effect of drug (*F*_(2,53)_ = 28.48, *p* < 0.001) and effect of duration (*F*_(1,53)_ = 24.18, *p* < 0.001), but no effect of subject (*F*_(53,53)_ = 0.77, *p* = 0.826). *Post hoc* results reveal a difference of diestrus period length during treatment between vehicle and formestane (*t*_(106)_ = 7.47, *p* < 0.001) and vehicle and letrozole (*t*_(106)_ = 5.61, *p* < 0.001), but no difference between formestane and letrozole (*t*_(106)_ = 0.62, *p* = 0.901). There were no differences in diestrus length between groups before treatment (Supplementary Table S3).

Aromatase inhibition decreased uterine weights compared to proestrus uteri (Figure 2C). There was a significant difference in uterine *indices* (F*_(3,25)_ = 31, *p* < 0.001, Brown-Forsythe ANOVA). *Post hoc* results reveal differences in uterine indices between proestrus and estrus (*t*_(9.7)_ = 5.0, *p* = 0.003), proestrus and formestane (*t*_(13)_ = 4.7, *p* = 0.003), proestrus and letrozole (*t*_(7.1)_ = 11, *p* < 0.001), estrus and letrozole (*t*_(17)_ = 9.2, *p* < 0.001), and formestane and letrozole (*t*_(16)_ = 6.5, *p* < 0.001). Notably, there was no difference in uterine indices between estrus and formestane mice (*t*_(23)_ = 0.22, *p* > 0.999, Supplementary Table S4).

Serum estradiol was quantified using ELISA. Because formestane, with a steroid-like structure, was recognized by the anti-estradiol antiserum, we quantified the hormone levels only in samples from letrozole treated mice. There were differences in serum estradiol-IR (*F*_(4,21)_ = 5.82, *p* = 0.003, ordinary one-way ANOVA, Figure 2D) between male vehicle and male letrozole (*t*_(21)_ = 3.16, *p* = 0.028), proestrus vehicle and estrus vehicle (*t*_(21)_ = 3.18, *p* = 0.027), and a near difference between proestrus vehicle and female letrozole (*t*_(21)_ = 2.63, *p* = 0.090, additional *post hoc* comparisons described in Supplementary Table S5). However, there was no difference between male and proestrus estradiol (*t*_(21)_ = 0.22, *p* > 0.999). In female mice, there was a positive correlation between serum estradiol-IR and uterine index across proestrus, estrus, and letrozole treatment (Pearson R^2^ = 0.218, *p* = 0.014, Figure 2E). Of note, nearly all apparent estradiol concentrations were below the lower detection limit of the assay (3 pg/ml).

To improve the assay, we extracted serum prior to ELISA. Extraction increased average concentrations and estradiol levels differed across groups (F*_(4,8.05)_ = 8.05, *p* = 0.007, Figure 2F; Brown-Forsythe ANOVA). Though these* post hoc* comparisons did not reach the threshold for significance, letrozole tended to decrease estradiol-IR, proestrus levels tended to be higher than estrus, and male levels were below female (Supplementary Table S5). Together, these findings indicate that aromatase inhibition, which protects male and female mice from MAS, reduces systemic estradiol.

### Systemic aromatase inhibition decreases ELISA-measured hippocampal estradiol-immunoreactivity, yet the compound is not recognized as estradiol on mass spectrometry

**Figure 3:**
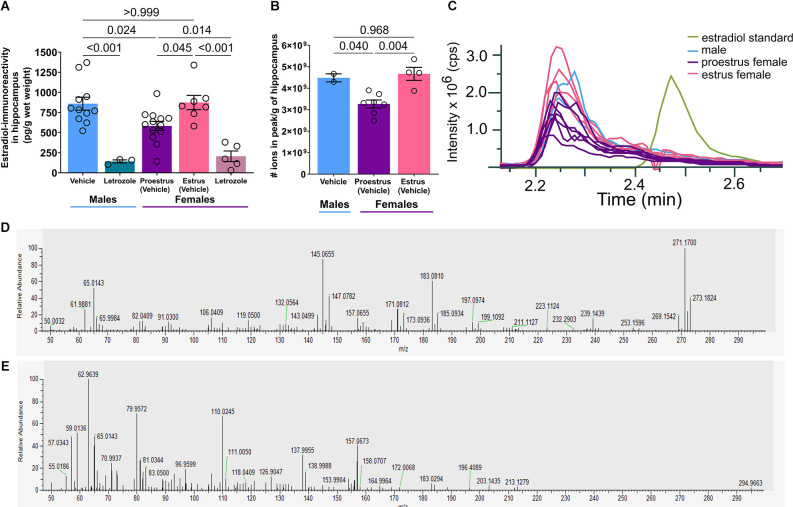
Systemic aromatase inhibition decreases hippocampal, ELISA-measured estradiol-immunoreactivity but estradiol was not detected by mass spectrometry. **(A)** Estradiol-immunoreactivity in the hippocampus (pg/g of hippocampus wet weight) as measured by estradiol ELISA. Letrozole reduces hippocampal estrogen. Estradiol in proestrus female hippocampus is lower than estradiol in male or estrous female hippocampus (*n* = 3–13/group). **(B)** The number of ions in the measured peaks (m/z 271.17) normalized to g of the hippocampus. Again, concentration in proestrus female hippocampus is lower than in male or estrous female hippocampus (*n* = 2–7/group). **(C)** The m/z 271.17 peak in hippocampus samples elute at 2.24 min while a 17β-Estradiol standard elutes at 2.47 min, indicating that these peaks are not 17β-Estradiol. **(D)** MS/MS spectrum of the [M-H]- ion (m/z 271.1704) for a 1:1 mixture of deuterated-labeled and unlabeled estradiol standards. **(E)** MS/MS spectrum of the [M-H]- ion (m/z 271.1704) for an unknown compound identified in a male hippocampus. Post test p-values are provided above the corresponding comparisons. Individual points represent individual mice. Data are presented as mean ± SEM.

To test if **hippocampal** estradiol, reportedly high in males and proestrus females (Kato et al., 2013; Hojo and Kawato, 2018; but see Caruso et al., 2013; Marbouti et al., 2020) enables MAS-induced memory problems, we quantified hippocampal estradiol across sex, cycle, and aromatase inhibition using ELISA. Letrozole reduced apparent hippocampal estradiol-IR in both sexes, however, levels were surprisingly lower in proestrus females compared to estrous females and males (*F*_(4,34)_ = 13.84, *p* < 0.001, Figure 3A, ordinary one-way ANOVA).* Post-hoc* tests revealed differences in hippocampal estradiol-IR between male vehicle and male letrozole (*t*_(34)_ = 5.05, *p* < 0.001), male vehicle and proestrus vehicle (*t*_(34)_ = 3.09, *p* = 0.024), proestrus vehicle and estrus vehicle (*t*_(34)_ = 2.84, *p* = 0.045), proestrus vehicle and female letrozole (*t*_(34)_ = 3.28, *p* = 0.014), and estrus vehicle and female letrozole (*t*_(34)_ = 5.23, *p* < 0.001), but no difference between male vehicle and estrus vehicle (*t*_(34)_ = 0.13, *p* > 0.999, Supplementary Table S6).

To validate hippocampal estradiol levels, we analyzed similarly processed tissue by mass spectrometry and detected peaks with the expected molecular weight of 17β-estradiol (271.17 g/mol). Quantifying measured ions/g of hippocampus, concentrations in female proestrus were again lower compared to male and estrous females (one-way ANOVA: *F*_(2,10)_ = 11.40, *p* = 0.003, Figure 3B). *Post-hoc* tests revealed a difference in concentration between male and proestrus (*t*_(10)_ = 3.00, *p* = 0.040) and proestrus and estrus (*t*_(10)_ = 4.42, *p* = 0.004), but no difference between male and estrus (*t*_(10)_ = 0.42, *p* = 0.968, Supplementary Table S6).

However, further analyses indicated that the peak identified in hippocampus was not estradiol. Elution times differed: 2.24 min for the compound, 2.47 min for 17β-estradiol standard (Figure 3C). This unknown peak was not identified in extracted water or estradiol samples. Given the identical molecular weight, we hypothesized that the compound might be the 17β-estradiol isomer: 17α-estradiol (Toran-Allerand et al., 2005). While it was difficult to distinguish enantiomer peaks without a chiral column or derivatization, the hippocampal peak still eluted earlier, suggesting that the compound detected here, and presumably through ELISA, was neither 17β-estradiol nor 17α-estradiol. We additionally examined MS/MS profiles of the estradiol standard vs. hippocampus (Figures 3D,E). Based on the disparate fragmentation patterns of estradiol standard and hippocampus, we conclude that these are not the same compound.

Therefore, we conclude that available commercial methodologies identify a compound in hippocampus that is not estradiol. Intriguingly, aromatase inhibition reduces its levels, yet its identity remains to be established.

## Discussion

Here we confirm that multiple acute concurrent stresses (MAS) disrupt hippocampus-dependent memory in male mice, and in females stressed during proestrus (Figure 1B; Chen et al., 2016; Hokenson et al., 2021). High levels of estrogens are required in both sexes, as reducing estrogens by inhibiting aromatase prevents MAS from disrupting memory (Figure 1B). These findings are surprising for both sexes. First, they support a deleterious role of high levels of estrogens in females. Second, they suggest a role for hippocampal estrogens in males. Whereas hippocampal estradiol is reportedly high (Hojo et al., 2009; Kato et al., 2013; Hojo and Kawato, 2018) there has been little work on its putative role. Here we suggest the novel notion that hippocampal estrogens in males may act to repress stressful memories.

We previously established that MAS-induced memory disruption and dendritic spine loss in males require convergent activation of corticotropin releasing hormone receptor 1 (CRHR1) and glucocorticoid receptor (GR; Chen et al., 2008, 2016). As high levels of estrogens are required for memory disruption in males and females (Figure 1B), these stress-induced disruptions may rely on the synergistic activation of estrogen receptors with CRHR1 and GR. Concurrent activation of these receptors, which converge on RhoA-pCofilin signaling (Chen et al., 2008, 2013; Kramár et al., 2009a, b, 2013), may destabilize dendritic spines. Alternatively, estrogens may influence the levels or activity of CRH or glucocorticoids. Indeed, a potential role for estradiol in augmenting CRH expression has been identified (Lalmansingh and Uht, 2008; Qi et al., 2020).

Here we tested the potential role of high estrogens in MAS-induced memory deficits using the aromatase blocker formestane. While estradiol-IR reductions with letrozole were generally quite large (Figures 2D–F, Figures 3A), formestane-induced reductions may be more modest. Indeed, formestane exerts less aromatase inhibition (84%–93%) than letrozole (>98%; Lønning, 2003), and likewise less potently reduces plasma estrogens (Jones et al., 1992; Geisler et al., 2002). Nevertheless, the pronounced effects of formestane on cycling and uterine weights (Figures 2B,C) and reported reduction in circulating estradiol with similar doses (Yue et al., 1995; Nißlein and Freudenstein, 2007) suggest that estrogens were significantly reduced. While estradiol, especially originating from the hippocampus, is critical for memory in female and male mice (Martin et al., 2003; Tuscher et al., 2016; Marbouti et al., 2020) control formestane treated females had only a mild reduction in preference for the moved object compared to control vehicle treated females (Figure 1B), suggesting that reduction of estrogens was incomplete. Alternatively, it is possible that week-long aromatase inhibition could increase testosterone or have direct androgenic properties (Séralini and Moslemi, 2001). While this may propose a protective role of androgens, our current work using estrogen receptor blockers suggest estrogen receptor activation plays a direct role in MAS-induced memory impairment.

ELISA is widely used in animal and clinical research due to its relatively low cost, convenience, high throughput, and safety compared to radioactive assays (Sakamoto et al., 2018). However, several issues were found here. Estradiol ELISAs have difficulties quantifying low concentrations, such as in males, ovariectomized, or aromatase-inhibited animals (Hsing et al., 2007; Huhtaniemi et al., 2012; Schumacher et al., 2015; Niravath et al., 2017). We have used the Calbiotech ELISA to distinguish proestrus and estrous female unextracted serum (Hokenson et al., 2021). This was accomplished, though nearly all values were below the lower limit of detection (Figure 2D). These low values are not unusual given low mice serum levels (Nilsson et al., 2015; Handelsman et al., 2020). Surprisingly, male serum levels were unexpectedly high, similar to proestrus females (Figure 2D). Purification may increase estradiol-IR by removing interfering substances (Chao et al., 2011; Tuscher et al., 2016; Boyaci et al., 2020; Krentzel et al., 2020; Proaño et al., 2020). Indeed, extracting serum prior to ELISA enhanced estradiol-IR and distinguished male and female values (Figure 2F) but were higher than expected of mouse serum (Nilsson et al., 2015; but see Marbouti et al., 2020, analyzed by ELISA). Artificially high estradiol-IR could be due to the tendency for ELISA to overestimate levels (McNamara et al., 2010). Indeed, the Calbiotech plate may not distinguish between intact vs. ovariectomized mice (Haisenleder et al., 2011).

Regarding hippocampal estradiol, we expected lower levels in estrus females vs. proestrus females and males (Kato et al., 2013). Instead, ELISA found the lowest estradiol-IR in proestrus females (Figure 3A). Therefore, we turned to mass spectrometry and given the high levels of apparent estradiol-IR by ELISA (Figure 3A), anticipated that estradiol levels would be quantifiable without derivatization. Indeed, we found peaks of the expected molecular weight in our samples and, similar to ELISA, a reduction in proestrus female levels (Figure 3B). However, the peaks in the hippocampus were not estradiol based on different retention times and MS/MS profiles (Figures 3C–E). Only hippocampi were analyzed through mass spectrometry, so it is unknown whether the same unknown peak is found in serum, or if ELISA and mass spectrometry detect the same compound. These factors lead us to conclude that ELISA-measured estradiol values should be considered with extreme caution (Schumacher et al., 2015).

We probably failed to detect estradiol with our current mass spectrometry approach because estradiol quantities were too low or extraction procedures insufficient. While large quantities of tissue estradiol have been quantified without derivatization (McNamara et al., 2010), other groups have successfully detected small quantities through estradiol-specific derivatization (Kato et al., 2013; Jalabert et al., 2022) or signal-enhancing additives (Lozan et al., 2017).

In conclusion, aromatase inhibition protects male and female mice from the memory impairing effects of MAS, suggesting that high levels of estrogens are required for MAS to disrupt memory. Future studies will probe the specific estrogen receptors involved. Additionally, studies are clearly warranted to definitively measure estradiol in murine hippocampus.

## Data Availability Statement

The raw data supporting the conclusions of this article will be made available by the authors, without undue reservation.

## Ethics Statement

The animal study was reviewed and approved by University of California—Irvine’s Institutional Animal Care and Use Committee (IACUC).

## Author Contributions

RH and TZB designed the research. RH, YA, AS, and SJ conducted the research. RH, AS, YA, SJ, CJ, and TZB analyzed the data. RH and TZB wrote and edited the article. All authors contributed to the article and approved the submitted version.

## Funding

This study was supported by NIH R01 MH073136 (TZB), P50 MH096889 (TZB), and T32 MH119049-02 (RH).
