# Supplementary material for: Sex-dependent effects of multiple acute concurrent stresses on memory: a role for hippocampal estrogens
Source: Front Behav Neurosci. 2022 Sep 8;16:984494. doi: 10.3389/fnbeh.2022.984494 (PMC9492881; doi:10.3389/fnbeh.2022.984494)
Supplement: Supplementary file 1 [file Data_Sheet_1.docx]

Supplementary Material

Here we include supplementary tables providing details about the Calbiotech ELISA kit and the post hoc test results of our statistical analyses described in the main text.

Table S1. **Details of the Calbiotech Mouse/Rat Estradiol ELISA Kit (ES180S-100).** Performance and cross-reactivity are reported from data collected by the Ligand Core (Haisenleder et al., 2011)

| Kit characteristics | |
| --- | --- |
| Sample volume | 25µL |
| Functional sensitivity | 3pg/mL |
| Assay dynamic range | 0-300pg/mL |
| Intraassay precision | 3.1% |
| Interassay precision | 9.9% |
| Average % Recovery | 167-175% |
| Correlation with GC/MSMS | r^2^ = 0.969 |
|  |  |
|  | |
| Reported compound cross reactivity | |
| Compound | Cross reactivity [%] |
| Progesterone | 0.0002 |
| Androstenedione | 0.0001 |
| Testosterone | 0.0002 |
| Cortisol | 0.0001 |
| Other endogenous steroids | <0.0001 or undetectable |

Table S2. **Post hoc test results for novel/familiar location ratio during the OLM test session.** Sidak’s multiple comparisons test.

| Comparison | | Mean 1 | Mean 2 | Mean Diff | SE of diff. | t | DF | Adjusted P value |
| --- | --- | --- | --- | --- | --- | --- | --- | --- |
| Vehicle control males | Formestane control males | 2.02 | 2.03 | -0.018 | 0.360 | 0.05 | 54 | >0.999 |
| Vehicle MAS males | Formestane MAS males | 1.05 | 2.24 | -1.19 | 0.338 | 3.51 | 54 | 0.004 |
| Vehicle control females | Formestane control females | 2.17 | 1.58 | 0.591 | 0.371 | 1.59 | 54 | 0.393 |
| Vehicle MAS females | Formestane MAS females | 1.06 | 2.22 | -1.16 | 0.347 | 3.35 | 54 | 0.006 |

Table S3. **Post hoc test results for time in diestrus.** Percent of observation period spent in the diestrus phase of the estrous cycle is quantified before and during treatment with aromatase inhibitors. Sidak’s multiple comparisons test.

| Comparison | | Pred. (LS) Mean 1 | Pred. (LS) Mean 2 | Pred. (LS) Mean Diff | SE of diff | N1 | N2 | t | DF | Adjusted P value |
| --- | --- | --- | --- | --- | --- | --- | --- | --- | --- | --- |
| Before treatment | |  |  |  |  |  |  |  |  |  |
| Vehicle | Formestane | 41.01 | 50.72 | -9.705 | 5.241 | 25 | 24 | 1.85 | 106 | 0.187 |
| Vehicle | Letrozole | 41.01 | 46.94 | -5.931 | 7.842 | 25 | 7 | 0.76 | 106 | 0.835 |
| Formestane | Letrozole | 50.72 | 46.94 | 3.774 | 7.878 | 24 | 7 | 0.48 | 106 | 0.951 |
| During treatment | |  |  |  |  |  |  |  |  |  |
| Vehicle | Formestane | 39.90 | 79.05 | -39.14 | 5.241 | 25 | 24 | 7.47 | 106 | <0.001 |
| Vehicle | Letrozole | 39.90 | 83.93 | -44.02 | 7.842 | 25 | 7 | 5.61 | 106 | <0.001 |
| Formestane | Letrozole | 79.05 | 83.93 | -4.883 | 7.878 | 24 | 7 | 0.62 | 106 | 0.901 |

Table S4. **Post hoc test results for uterine indices.** Uterine indices (uterine wt (g)/body wt (g) * 100) are compared across estrous cycle phase and aromatase inhibitor treatment. Dunnett’s T3 multiple comparisons test.

| Comparison | | Mean 1 | Mean 2 | Mean Diff | SE of diff. | N1 | N2 | t | DF | Adjusted P value |
| --- | --- | --- | --- | --- | --- | --- | --- | --- | --- | --- |
| Proestrus vehicle | Estrus vehicle | 0.42 | 0.27 | 0.15 | 0.030 | 7 | 13 | 5.0 | 9.7 | 0.003 |
| Proestrus vehicle | Formestane | 0.42 | 0.26 | 0.15 | 0.033 | 7 | 14 | 4.7 | 13 | 0.003 |
| Proestrus vehicle | Letrozole | 0.42 | 0.12 | 0.30 | 0.027 | 7 | 7 | 11 | 7.1 | <0.001 |
| Estrus vehicle | Formestane | 0.27 | 0.26 | 0.01 | 0.025 | 13 | 14 | 0.22 | 23 | >0.999 |
| Estrus vehicle | Letrozole | 0.27 | 0.12 | 0.15 | 0.016 | 13 | 7 | 9.2 | 17 | <0.001 |
| Formestane | Letrozole | 0.26 | 0.12 | 0.14 | 0.022 | 14 | 7 | 6.5 | 16 | <0.001 |

Table S5. **Planned post hoc comparisons for serum estradiol concentrations.** Concentrations (pg/mL) are quantified by ELISA using serum directly onto the plate or using serum that was first extracted. Samples are compared by sex/cycle and each drug treatment is compared within sex. Sidak’s multiple comparisons test for unextracted serum and Dunnett’s T3 multiple comparisons test for extracted serum.

| Comparison | | Mean 1 | Mean 2 | Mean Diff | SE of diff. | N1 | N2 | t | DF | Adjusted P value |
| --- | --- | --- | --- | --- | --- | --- | --- | --- | --- | --- |
| Unextracted serum | |  |  |  |  |  |  |  |  |  |
| Male vehicle | Male letrozole | 2.118 | 0.055 | 2.063 | 0.652 | 3 | 3 | 3.16 | 21 | 0.028 |
| Male vehicle | Proestrus vehicle | 2.118 | 2.235 | -0.116 | 0.541 | 3 | 8 | 0.22 | 21 | >0.999 |
| Male vehicle | Estrus vehicle | 2.118 | 0.965 | 1.153 | 0.541 | 3 | 8 | 2.13 | 21 | 0.242 |
| Proestrus vehicle | Estrus vehicle | 2.235 | 0.966 | 1.269 | 0.340 | 8 | 8 | 3.18 | 21 | 0.027 |
| Proestrus vehicle | Female letrozole | 2.235 | 0.948 | 1.287 | 0.489 | 8 | 4 | 2.63 | 21 | 0.090 |
| Estrus vehicle | Female letrozole | 0.966 | 0.948 | 0.018 | 0.483 | 8 | 4 | 0.04 | 21 | >0.999 |
|  |  |  |  |  |  |  |  |  |  |  |
| Extracted serum | |  |  |  |  |  |  |  |  |  |
| Male vehicle | Male letrozole | 3.54 | 3.68 | -0.137 | 0.378 | 7 | 2 | 0.36 | 6.64 | 0.999 |
| Male vehicle | Proestrus vehicle | 3.54 | 106.4 | -102.8 | 35.32 | 7 | 9 | 2.91 | 8.00 | 0.096 |
| Male vehicle | Estrus vehicle | 3.54 | 9.99 | -6.453 | 2.063 | 7 | 7 | 3.13 | 6.39 | 0.094 |
| Proestrus vehicle | Estrus vehicle | 106.4 | 9.99 | 96.28 | 35.37 | 9 | 7 | 2.73 | 8.05 | 0.125 |
| Proestrus vehicle | Female letrozole | 106.4 | 4.74 | 101.6 | 35.32 | 9 | 2 | 2.88 | 8.00 | 0.100 |
| Estrus vehicle | Female letrozole | 9.99 | 4.74 | 5.25 | 2.07 | 7 | 2 | 2.54 | 6.40 | 0.191 |

Table S6. **Planned post hoc comparisons for hippocampal estradiol concentration.** Concentrations are quantified by ELISA (pg/g hippocampus wet weight) or mass spectrometry (ions/g hippocampus wet weight). Samples are compared by sex/cycle and each drug treatment is compared within sex. Sidak’s multiple comparisons test.

| Comparison | | Mean 1 | Mean 2 | Mean Diff | SE of diff. | N1 | N2 | t | DF | Adjusted P value |
| --- | --- | --- | --- | --- | --- | --- | --- | --- | --- | --- |
| Estradiol ELISA | |  |  |  |  |  |  |  |  |  |
| Male vehicle | Male letrozole | 859.9 | 141.6 | 718.2 | 142.3 | 11 | 3 | 5.05 | 34 | <0.001 |
| Male vehicle | Proestrus vehicle | 859.9 | 583.2 | 276.6 | 89.5 | 11 | 13 | 3.09 | 34 | 0.024 |
| Male vehicle | Estrus vehicle | 859.9 | 874.1 | -14.2 | 105.6 | 11 | 7 | 0.13 | 34 | >0.999 |
| Proestrus vehicle | Estrus vehicle | 583.2 | 874.1 | -290.9 | 102.4 | 13 | 7 | 2.84 | 34 | 0.045 |
| Proestrus vehicle | Female letrozole | 583.2 | 205.6 | 377.6 | 115.0 | 13 | 5 | 3.28 | 34 | 0.014 |
| Estrus vehicle | Female letrozole | 874.1 | 205.6 | 668.5 | 127.9 | 7 | 5 | 5.23 | 34 | <0.001 |
|  | |  |  |  |  |  |  |  |  |  |
| Mass spectrometry | |  |  |  |  |  |  |  |  |  |
| Male vehicle | Proestrus vehicle | 4485981539 | 3268165138 | 1217816401 | 406303221 | 2 | 7 | 3.00 | 10 | 0.040 |
| Male vehicle | Estrus vehicle | 4485981539 | 4670987475 | -185005936 | 438857637 | 2 | 4 | 0.42 | 10 | 0.968 |
| Proestrus vehicle | Estrus vehicle | 3268165138 | 4670987475 | -1402822337 | 317621838 | 7 | 4 | 4.42 | 10 | 0.004 |

# Literature Cited

Haisenleder, D. J., Schoenfelder, A. H., Marcinko, E. S., Geddis, L. M., & Marshall, J. C. (2011). Estimation of estradiol in mouse serum samples: Evaluation of commercial estradiol immunoassays. *Endocrinology*, *152*(11), 4443–4447. https://doi.org/10.1210/en.2011-1501
